# Case report: An outbreak of viral conjunctivitis among the students and staff of visually impaired school, Tamil Nadu, India, 2020

**DOI:** 10.3389/fpubh.2022.978200

**Published:** 2022-08-05

**Authors:** Yazhini Madurapandian, Polani Rubeshkumar, Mohankumar Raju, Aishwarya Janane, Parasuraman Ganeshkumar, T. S. Selvavinayagam, Prabhdeep Kaur

**Affiliations:** ^1^ICMR-National Institute of Epidemiology, Chennai, Tamil Nadu, India; ^2^Directorate of Public Health and Preventive Medicine, Chennai, Tamil Nadu, India; ^3^Shri Sarradha Eye Hospital, Pudukkottai, Tamil Nadu, India

**Keywords:** conjunctivitis, viral conjunctivitis, disease outbreak, India, risk factors

## Abstract

**Introduction:**

On February 2, 2020, the head of a visually impaired school notified similar eye symptoms among the students. We investigated the cluster to confirm the diagnosis, identify potential exposures, and propose recommendations.

**Methodology:**

We defined a case as redness/watering/discharge from any eye among the students and staff, January–February 2020. We actively searched for the cases and calculated attack rates. We drew epicurve by date of symptoms onset. We conducted a retrospective cohort study of students and staff. We collected data on potential exposures and calculated Risk Ratio (RR), 95% Confidence Interval (95%CI), and Population Attributable Risk (PAR). We sent a conjunctival swab of the three cases for microbiological analysis.

**Results:**

We diagnosed the cases as acute conjunctivitis and identified 39 (76%) cases among 51 individuals. All the 39 cases reported watering and redness; 28 (72%) and 12 (31%) reported eye pain and discharge, respectively. The median age of the case was 11 years (range: 6–48 years). The attack rate didn't differ significantly between males [77% (20/26)] and females [76% (19/25), *p* = 0.9]. The attack rate was higher among the students [86%, (38/44)] than staffs [14%, (1/7), *p* = <0.01]. Contact with a case [RR = 2.5, 95%CI = 1.3–4.8, PAR = 51%] and staying inside campus [RR = 6.0, 95%CI = 1.0–37.3, PAR = 81%] were associated with the acute conjunctivitis outbreak. All the three conjunctival swabs were negative for bacterial growth.

**Conclusion:**

Close contact with the case and staying inside the campus led to the outbreak of acute conjunctivitis among the students and staff of the visually impaired school.

## Introduction

Conjunctivitis is one of the most common causes of red-eye and affects patients of all ages and socioeconomic classes. Viral conjunctivitis is responsible for the majority of infectious conjunctivitis, accounting for up to 75% of cases (1). Viral conjunctivitis is a highly contagious acute conjunctival infection usually caused by adenovirus (1). Epidemic keratoconjunctivitis usually results from adenovirus serotypes Ad 5, 8, 11, 13, 19, and 37 and tends to cause severe conjunctivitis (1). The diagnosis of conjunctivitis is predominantly clinical, and laboratory investigations are not indicated in all cases unless the symptoms did not subside. The conjunctivitis outbreaks are not uncommon (2–4). Such outbreaks are frequently linked to people congregation settings like hostels, classrooms, shared accommodations (2–4). On February 2, 2020, the head of a school for visually impaired children notified about the occurrence of similar eye complaints among a few students. A local team consisted of an ophthalmologist and epidemiologist to investigate the cluster of eye illnesses reported among students to confirm the diagnosis, identify the potential exposures and propose recommendations.

## Methodology

We conducted an outbreak investigation of a cluster of eye complaints among visually impaired students based on the field epidemiology steps of the outbreak investigation (5, 6). An ophthalmologist examined the reported case patients and provided inputs to formulate a case definition of this cluster. We defined a case as the occurrence of any of the following eye symptoms: redness, watering, discharge, foreign-body sensation in any of the eyes among the students and staff of the visually impaired school from January 20 to February 28, 2020. We actively searched for the cases among the students and staff meeting the case definition and line-listed them. We collected demographic and clinical symptoms data in the line list. We interviewed a few key informants like case-patients and staff of the visually-impaired school about the sequence of events and illnesses to generate a hypothesis. We collected conjunctival swabs from three case-patients for microbiological analysis. We conducted a retrospective cohort study of the students and staff of the visually impaired school to test the hypothesis. We collected data on potential exposures using a semi-structured data collection tool through interviews. We used Epi Info (Ver. 7.2) for data management and analysis (7).

We described the cases by date of symptom onset as epi-curve. We described the clinical symptoms reported by the case-patients as proportions. We calculated the median age of the detected case-patients with range. We calculated the attack rates by age, gender, student/staff and place of stay with appropriate denominators and expressed as per 100 persons. We compared the attack rates of conjunctivitis between the exposed and non-exposed groups to compute Risk Ratio (RR) with 95% Confidence Interval (95%CI) and Population Attributable Risk (PAR). We considered *p*-value <0.05 as statistically significant.

## Results

There were 51 individuals in the visually-impaired school; among them, 44 (86%) were students, and 7 (14%) were the staff. The median age of the students was 11 years and ranged between 6 and 13 years. Among the 44 students, 24 (55%) were males, and 38 (86%) were staying in the hostel within the school campus. The median age of the staff was 42 years and ranged between 34 and 48 years. Among the seven staff, five were females, and three were staying in the hostel.

**Figure 1:**
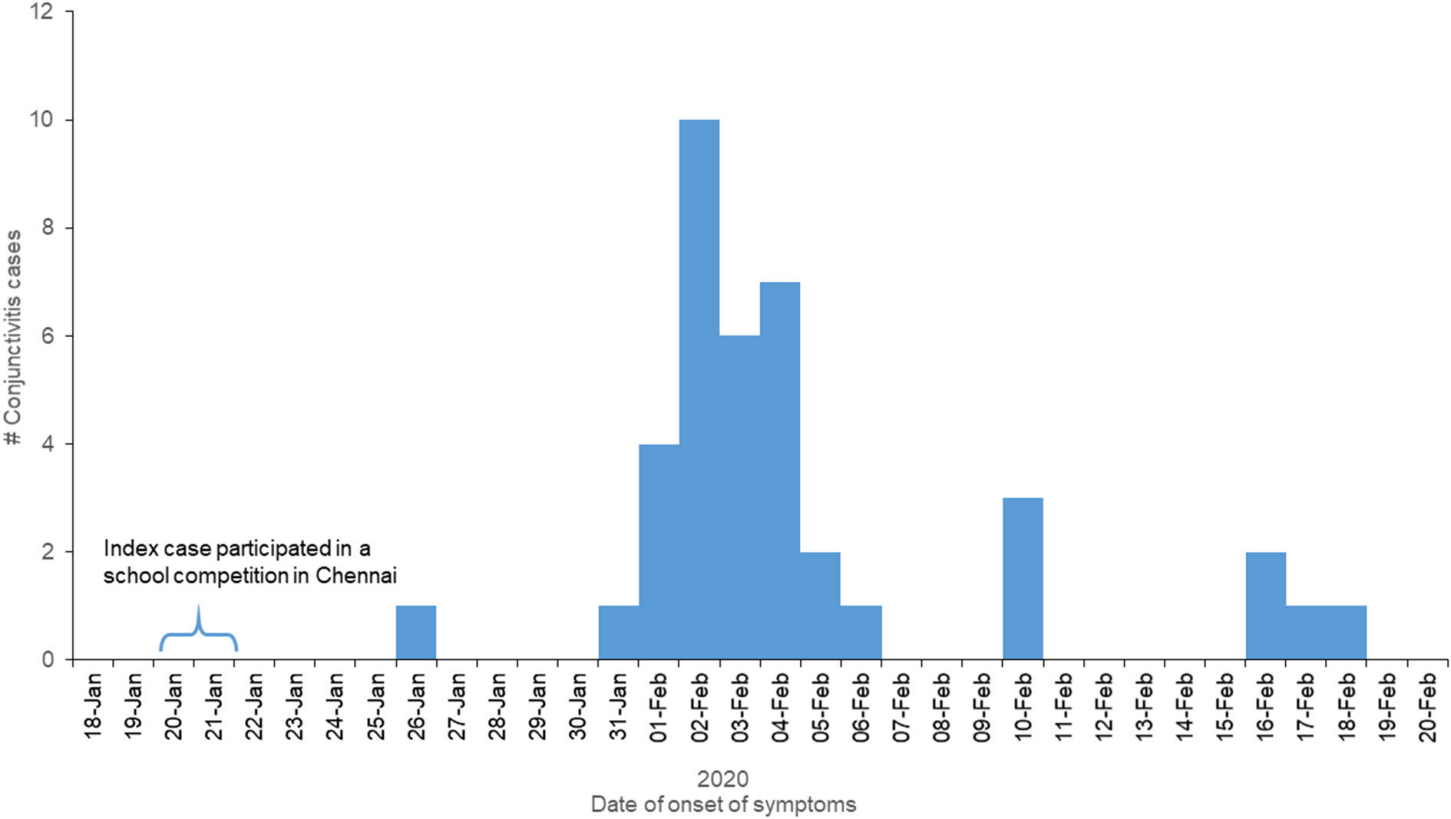
Acute conjunctivitis cases by date of onset of symptoms among the students and staff of the visually-impaired school, Tamil Nadu, India, Jan–Feb 2020.

The Ophthalmologist clinically diagnosed the identified cases as acute conjunctivitis. Among the 51 individuals of the visually impaired school, we identified 39 (76%) cases in which 38 (97%) were students. The median age of the case-patients was 11 years (Range: 6–48 years). The cases were reported between January 26, 2020, and February 18, 2020 (Figure 1). The pattern of the epi-curve suggested a person-to-person transmission (Figure 1). The key informants' interviews revealed that the index case had an outstation travel history and attended an inter-school competition event in Chennai, India, on Jan 20 and 21, 2020 and developed symptoms 5 days post-event. Among the 39 cases, 20 (51%) were males, and 38 (97%) stayed in the hostel. All the 39 case-patients reported watering and redness of both eyes. Eye pain and discharge in either of the eyes were reported by 28 (72%) and 12 (31%) case-patients, respectively. Almost all the cases developed the second eye infection within the 48 h of development of the first eye symptom.

The overall attack rate of acute conjunctivitis was 76% (39/51). The attack rate didn't differ significantly between males [77%, (20/26)] and females [76%, (19/25), *p* = 0.9]. The attack rate was higher among the students [86%, (38/44)] than staffs [14%, (1/7), *p* = <0.001]. Based on the descriptive epidemiology findings and key informant interviews, we hypothesized the following potential exposures for this outbreak: male gender, hostel resident and close contact with a case.

We included all 51 individuals in the retrospective cohort study. We observed that the risk for acute conjunctivitis was 2.5 times [RR = 2.5, 95%CI = 1.3–4.8, PAR = 51%] higher among those who had contact with the case [94%, (33/35)] than did not [38%, (6/16)]. We also found that being a resident of the hostel [86%, (38/44)] was six times [RR = 6.0, 95%CI = 1.0–37.3, PAR = 81%] higher risk for acute conjunctivitis than non-resident of the hostel [14%, (1/7); Table 1). All three conjunctival swabs were negative for bacterial growth. We treated all the cases and followed up till their complete recovery. We managed the case-patients with analgesics, cold compresses, and artificial tears. All the cases recovered between 7 and 10 days from the date of onset of symptoms. We implemented quarantine and educated personal hygiene measures.

**Table 1:** Attack rate of acute conjunctivitis by different exposures among the students and staff of a visually-impaired school, Tamil Nadu, India, Jan–Feb 2020.

	**Attack rate of acute conjunctivitis**									
	**Exposed**			**Unexposed**						
	**#**	**Total**	**%**	**#**	**Total**	**%**	**Relative risk**	**95%CI**	***p*** **-Value**	**PAR %**
Male gender	20	26	77	19	25	76	1.0	0.7–1.4	1.0	0.6
Close contact with a case	33	35	94	6	16	38	2.5	1.3–4.8	0.00003	51
Staying in the hostel	38	44	86	1	7	14	6.0	1.0–37.3	0.0003	81
Poor hand hygiene	31	36	86	8	15	53	1.6	1.0–2.6	0.02	30

## Discussion

The outbreaks of conjunctivitis often go unreported in our routine disease surveillance system unless it draws the local media attention. Identifying the cluster of conjunctivitis and controlling the spread is vital to prevent a large community outbreak. We investigated an acute conjunctivitis outbreak in a visually impaired school with a hostel setting. In our investigation, a hoste l resident where the contact with the potential occurred was the primary source of infection. The congregation settings such as hostels, barracks, religious and social gatherings favor the transmission of disease spread through droplets (3, 8, 9). In this study, the index case, who attended the school competition with a travel history and transmitted the infection to their contacts. Isolation of the case after developing the clinical symptoms was not helpful at the time of investigation since the contacts were already exposed.

In this investigation, we could not identify the causative organism which caused the outbreak due to resource limitations. The most common causative organism of conjunctivitis is adenovirus, and it is diagnosed clinically (1). The gold standard test for adenovirus is virus isolation and cell culture (10). In developed countries, the laboratory confirmation of adenovirus is done by detecting viral DNA by polymerase chain reaction and rapid diagnostics kits (10, 11). However, these methods are expensive and are not available in India. We established epidemiological linkage between the cases and strength of association between exposure and conjunctivitis.

## Conclusion

Contact with an index case and staying in a closer congregation setting led to this outbreak of acute conjunctivitis among the students and staff of a visually impaired school. Education and awareness regarding early identification of symptoms, following personal hygiene measures and prompt isolation, would prevent such outbreaks in future in the similar settings. However, adopting such preventive measures among visually-impaired students with a closed hostel setting is challenging. We suggest the health education of the staff and caretaker of such challenging settings would identify the symptomatic individuals earliest.

## Data availability statement

The raw data supporting the conclusions of this article will be made available by the authors, without undue reservation.

## Author contributions

YM, PR, and AJ did the data collection. YM, PR, MR, and PG were involved in data analysis and visualization. PR, MR, PG, and PK drafted the manuscript. All the authors were involved in conceptualization, gave inputs, and accepted the final version of the manuscript.

## Conflict of interest

The authors declare that the research was conducted in the absence of any commercial or financial relationships that could be construed as a potential conflict of interest.

## Publisher's note

All claims expressed in this article are solely those of the authors and do not necessarily represent those of their affiliated organizations, or those of the publisher, the editors and the reviewers. Any product that may be evaluated in this article, or claim that may be made by its manufacturer, is not guaranteed or endorsed by the publisher.
